# Odontomes

**Published:** 1888-01

**Authors:** J. Bland Sutton

**Affiliations:** Hunterian Professor; late Erasmas Wilson Lecturer on Pathology, Royal College of Surgeons; Honorary Member of the Odontological Society of Great Britain.


ARTICLE IV.

### ODONTOMES.

BY J. BLAND SUTTON, F. R. C. S.

Hunterian Professor; late Erasmus Wilson Lecturer on Pathology, Royal College of Surgeons; Honorary Member of the Odontological Society of Great Britain.]

In the most extended sense an odontome may be defined as *a neoplasm composed of dental tissues (enamel, dentine, and cementum), in varying proportions and different degrees of development, arising from tooth-germs, or teeth still in the process of growth.*

It is customary to restrict the term, at least from the clinical point of view, to those hard tumours found in the jaws, composed of fully developed enamel, dentine, cementum, or varieties of these tissues. Nevertheless, the careful observations made by several workers during the past few years render a reconsideration of the matter imperative; for the term should apply not only to solid dental tumours, but to certain cystic forms as well. This extension of the term is of some importance, for we can then include several curious aberrations of tooth-development.

In the higher mammals three distinct parts are concerned in the formation of a tooth:—

1. The enamel organ, derived from the surface epithelium.
2. The dentine papilla.
3. The follicular wall, which, with the surrounding tissue, furnishes the cementum.

It would be obviously convenient if we could base a rigid classification of odontomes upon the embryological history of a tooth. This method, which has been the one mainly adopted since its introduction by Broca in 1869, has not been attended with success.

In the present essay I shall venture to classify odontomes in four groups. The novelty of the method of classification proposed consists in including certain cystic formations and fibrous tumours of the jaws among odontomes. How far this course is justifiable will be seen by the evidence which will be placed before you. The accompanying table represents in a complete form the mode of classification suggested. One of the chief features in this proposed method of arranging these tumours is, that the mode of genesis of the odontome, as well as its structure, is taken into consideration.

CLASSIFICATION OF ODONTOMES.

A. *Aberrations of the Enamel Organ*—

1. Epithelial Odontomes.
2. Calcified Epithelial Odontomes.

B. *Aberrations of the Follicle*—

1. Follicular Cysts.
2. Fibrous Odontomes.
3. Cementomata.

C. *Aberrations of the Papilla*—

1. Radicular Odontomes—
   *a.* Dentomata.
   *b.* Osteo-dentomata.
   *c.* Cementomata.

D. *Aberrations of the whole tooth-germ*—

Composite Odontomes.

E. *Anomalous Odontomes.*

A. *Aberrations of the Enamel Organ.*

*Epithelial Odontomes.*—This name is applied to those tumours which probably have their origin in aberrations of enamel germs, either of teeth which, normally, should go on to full development, or, as Mr. Eve suggests, from epithelial ingrowths around the dental alveoli, some of which may possibly be regarded as the representatives of teeth long since suppressd in the process of evolution.

To Mr. Eve belongs the credit of drawing attention to the true nature of this kind of odontome, under the name of multilocular cystic epithelial tumor. Details of some thirty cases are now known, from which the following characters have been drawn :—

Most of the patients come under observation at the age of twenty years, although the disease may occur at any period from infancy to old age. More commonly the lower jaw is affected, and the molar region is usually, though not exclusively, involved.

In typical specimens the tumour displays on section a congeries of cysts, in size very various, but they rarely exceed an inch in diameter. The cysts are separated by thin, fibrous septa, in some cases by osseous tissue. The cavities are, as a rule, filled with mucoid fluid of a brownish colour. The growing portions of the tumour are of a reddish-brown colour, not unlike that of a myeloid sarcoma.

Histologically, these tumours are composed of branching and anastomosing rods or columns of epithelium, portions of which form alveoli. The stroma is composed of fibrous tissue; when abundant, embryonic tissue in various stages is present. The cells occupying the alveoli vary; the outlay layer may be columnar, whilst the central cells degenerate and give rise to a reticulum of stellate cells resembling in structure the *stratum intermedium* of the enamel organ.

The naked eye appearance of these tumours is very characteristic. It is usual to find that in many of these cases, as in odontomes generally, absence of one or more of the teeth in the molar region. In the specimen represented, all the teeth in front of the molar were absent. The incisor shown in the figure belonged to the right side. The defect in the number of teeth in these cases supports the views also of Falkson and Bryck, who believe that, in some instances at least, these cysts have their origin in persistent portions of epithelium forming the enamel organ of developing teeth.

Mr. Eve's views concerning the origin of these odontomes from germs of teeth, or of suppressed teeth, has received considerable support from the observations of Malassez. This investigator has detected spherical, oval and cylindrical masses of epithelium within the periodontal membrane, and extending from the gum to the apex of the fang of a tooth. The epithelial collections are chiefly distributed below the necks of the teeth, and at the upper part of the root.

It is also satisfactory to find that Mr. Heath believes these views to be confirmed by clinical experience, and he adopts the term multilocular cystic epithelial tumour in preference to the older and very unsatisfactory name of cystic sarcoma. From a clinical, pathological, and evolutionary standpoint, I accept these views unreservedly, and feel that this mode of regarding tumours is a very distinct advance upon the usual narrow lines adopted in the study of human pathological anatomy.

*Calcified Epithelial Odontomes.*—We must now consider the relation existing between such tumours and the hard odontomes. It has already been mentioned that the septa in these cystic tumours, though usually fibrous, are occasionally composed of bone, and it certainly is easy to perceive that if the mass became in great part ossified we should obtain a hard odontome. On this head, Mr. Charles Tomes has stated that he has observed in at least one specimen of calcified odontome a structure which was such as would be produced by calcification of such tumours. It was for the most part made up of the products of calcifications of an infinitely branched enamel organ, there being little trace of dentine or of anything which appeared to have been derived from the calcification of a dentine pulp. A specimen which seems to belong to this form is one figured and described by Wedl.

Before dismissing aberrations of the enamel organ, mention must be made of the following, probably unique, specimen of a single cyst occupying the right side of a jaw, preserved in the Museum of the Royal College of Surgeons. It is thus described by Mr. Eve:—

"It contains no trace of a tooth, and the structure of its wall shows that it is not dentigerous or follicular, in the strict sense of the term, for it is lined by a thick layer of small round-celled epithelium. This may have originated from the expansion of a rudimentary enamel organ owing to the collection of fluid in its interior."

This cyst is of considerable interest, not only for the ætiological interpretation which may, with good reason, be applied to it, but because it illustrates the destructive effects of a central cystic growth upon the lower jaw. In this case the bony plates forming the angle and ramus of the maxilla are expanded into very thin laminæ; the condyle, detached from the ramus, is attached to the summit of the cyst by fibrous tissue; the coronoid process is in a similar condition, and the temporal muscle forms an exceedingly thin investment to the outer part of the cyst wall.

### B. *Aberrations of the Follicle.*

1. *Follicular Odontome.*—The term follicular is here adopted in preferance to the older adjective dentigerous, because the latter term has often been applied loosely to any cyst which bears teeth. The result of this inexactness has been to induce confusion, because all cysts which bear teeth are not dentigerous, even when they occur in connection with the jaws; for those curious cysts termed dermoid, and the heterogenous masses, called parasitic fœtuses, often contain teeth.

The true dentigerous cyst can only occur in connection with the jaws, and is to be regarded as a distension of the follicule in which the developing tooth is enclosed. If we can agree to apply the term *follicular* to such cysts, misapplication of the word is less likely to ensue.

An extended study of these cysts has led me to some interesting conclusions, for I think it will be possible to show that certain forms of odontomes of the hard variety arise from aberrations of this particular element of a tooth.

The characters of follicular cysts, as is well known, are the following:—

They arise in relation with teeth which have remained within the jaws—retained teeth; they are connected most commonly with those of the permanent set, but may affect also a temporary tooth; this, however, is rare. They are most frequently connected with the molars, and occur in the upper and lower jaws. In the latter situation they expand the bone and produce extensive deformity, tending the surgeon to believe that he has to deal with a solid tumour. In the upper jaw the cyst invades the antrum. In the simplest variety the cyst wall is composed of fibrous tissue, and the cavity is filled with glairy viscid fluid. Projecting into the cyst in most cases is the fang of an undeveloped tooth; sometimes, however, the tooth lies in the cavity of the cyst, and in rare instances the crown of the tooth is well formed, but the fang is imperfect.

Follicular cysts are liable to secondary changes; one of the most frequent is calcification of the cyst wall. All cysts of this nature which I have been able to examine presented this character. The amount of calcareous material varies; in some instances the wall is a shell of bone.

The best explanation yet offered concerning the ætiology of follicular cysts is that by Tomes, viz., that they arise from excessive formation around a retained tooth, that is between the enamel and the wall of the follicle, of a fluid which is normally found after the complete development of the enamel.

A follicular cyst may suppurate. This event seems to occur most frequently in the lower mammals.

2. *Fibrous Odontomes.*—In a developing tooth a portion of the connective tissue in which it is embedded is found to be denser and more vascular than the rest; it also presents a fibrillar arrangement. This condensed tissue is known as the tooth-sac, and when fully developed presents an outer firm wall and an inner looser layer of tissue. At the root of the tooth the follicle wall blends with the dentine papilla, and is indistinguishable from it. Before the tooth cuts the gum it is completely enclosed within this capsule.

The tooth-sac attains a large development in ruminants, especially the inner layer, in order to provide the large deposit of cementum for the crown of the tooth in this group. It must, however, be borne in mind that the follicular-sac is not alone responsible for the cementum.

This large development of the tooth-sac in ruminants is of some interest. In 1885 I described before this Society the skull of a goat which presented in each upper and lower jaw a thick-walled cyst developed in relation with a molar tooth. An examination of the wall of these cysts, aided by the microscope, showed it to be made up of lamellæ of fibrous tissue undergoing ossification. In the same paper I was able to describe a second case, also in a goat, and refer to one probably of the same nature recorded by Virchow. Since, an additional specimen has come under my notice, also in a goat.

This year I have had the good fortune to find another specimen in a Dasyure, which died in the Zoological Gardens. The skeleton was everywhere softened by rickets, the lower jaw being so flexible that it could be twisted as easily as gutta-percha. On examining the head, each upper jaw was found to be occupied by a tumour of the size of a walnut; each tumour was distinctly encapsuled and of a deep flesh-colour on section. It is not a little remarkable that in all cases of this form of odontome, the growths are symmetrical. This case differs from that of the goats inasmuch as the upper jaws alone are affected by the tumours. It is a curious fact, in so far as man is concerned, that in nearly all the recorded cases of odontomes the tumour occupied the lower jaw.

On microscopical examination the tumour was found to consist of lamellæ of dense fibrous tissue, enclosing a central softer portion in which the fangs of a molar tooth projected. In many parts of this dense capsule tracts of bony tissue were found. Near the centre of the mass the fibrous layers became attached to the neck of the tooth and seemed to blend with the periodontal membrane. The central softer part of the tumour contained numerous giant cells and embryonic connective tissue; in some of the more successful sections a direct continuity of this tissue with the pulp of the teeth was clearly made out.

The chief difference in this specimen and that of the goat previously described, consists in the circumstances that the tumour in the Dasyure was far less ossified than in the goat. Possibly the presence of rickets may help to explain the difference.

We must remember that Broca, under the term *odontomes embryoplastiques*, included certain cases of fibrous tumours in the upper and lower jaws, and certainly the specimens from the goats and opossum support this view. I am further convinced that several of the tumours described in Mr. Heath's work on the "Injuries and Diseases of the Jaws" are of this nature.

Other examples may be mentioned, but the above are sufficient to leave little doubt on the mind that a few fibrous tumours of the jaws are in reality odontomes in course of development.

3. *Cementomata.*—In this variety the bulk of the tumour is made up of cementum, often arranged in layers if the tumour be large.

The most typical cases of cementomata occur in horses, and may attain a large size, Their frequent occurrence in the ungalta generally need not surprise us when we remember the abundance of cementum present normally in the teeth of these mammals.

Broca has described specimens of this kind occurring in horses, in connection with the molar teeth, and similar cases have been placed on record by Rosseaux, Goubaux, and Magitot.

In 1871 Mr. Charles Tomes described in the "Transactions of this Society an odontome connected with the molar tooth of a horse. The tumour was five or six times larger than the tooth, and weighed ten ounces.

A careful consideration of such cases left a very strong impression on my mind when studied in connection with the tumours described in the jaws of the goats namely, that had the goats lived, the soft tumours in their maxillæ would have become completely ossified, and given rise to hard odontomes—in fact, cementomata.

Fortunately, whilst engaged in studying the tumour of the jaw in the Dasyure, and the thick-walled cysts, other specimens came to hand, which have enabled me to raise this view from the domain of probability. These specimens will now be considered in detail.

### c. *Aberrations of the Papilla.*

*Radicular Odontomes.*—This term is applied to odontomes which arise after the crown of the tooth has been completed and whilst the roots are in the process of formation. As the crown of the tooth, when once formed, is unalterable, it naturally follows that should the root develop an odontome, enamel cannot enter into its composition, which, for the most part, would consist of dentine and osteo-dentine in varying proportions, these two tissues being the result of the activity of the papilla.

When such a tumour consists mainly or entirely of dentine it may be termed a *radicular dentoma.* If osteo-dentine preponderates, then the tumour may be called a *radicular osteo-dentoma*; or if cementum, then it is a *radicular cementoma.* The terms in each instance clearly set forth the source of the tumour as well as indicating the structural characters.

Radicular odontomes, though very rare in man, are comparatively common in mammals, especially rodents, whose teeth grow from persistent pulps. It is impossible to decide the nature of the tissue composing them without seeking aid from the microscope. Examples of each form will now be given :—

1. *Radicular Dentomata.*—As has just been stated, this form of odontome occurs with greatest frequency in rodents, the purest forms occuring in marmots.

It may here be mentioned that odontomes in the lower mammals are by no means most frequent in the lower jaws, as in man. Not uncommonly they are multiple.

2. *Radicular Cementomata.*—Odontomes of this variety differ in no respect in their external characters from those just considered. As is the case with nearly all odontomes, suppuration and necrosis of the bone had taken place. The pus had travelled along the socket of the tooth, which was so large that the teeth moved loosely in the jaw.

On microscopic examination three-fourths of the tumour was found to be composed of cementum. Small patches of dentine were detected here and there throughout the mass, and dark interglobular spaces.

Professor Windle and Mr. Humphreys have recently described from the human subject a specimen which seems to belong to this variety. It occurred in a young man aged twenty-five. The odontome was situated in the lower jaw, on the right side, in the neighbourhood of the second molar tooth. After more than four months' excruciating pain, accompanied with profuse suppuration, life being several times despaired of, the odontome, seven months after its presence was first noticed, became liberated and fell into his mouth. The crown is fairly well formed, the labial surface being perfect, the lingual somewhat tuberculated. The roots are fused into an irregular mass. The under surface is irregular, and is at one point excavated into a hollow.

It is much to be regretted that it is impossible to obtain sections of this interesting tumour. As far as can be judged, the odontome consists of cementum overlaid in places with enamel.

It is far from the purposes of this paper to deal with the effects of odontomes, but the clinical history of the preceding case is very valuable, we read "his life being several times despaired of." In many of the recorded cases a train of severe symptoms, and in some, terrible mutilation from the surgeon, has been the result of these tumours. In one case the operative procedure was followed by death.

In the case of one of the marmots, in the porcupine, and in the agouti, I am convinced that death resulted from the profuse suppuration and necrosis set up by the odontomes. When we remember the serious trouble frequently caused by the cutting of a wisdom tooth, we can very well understand the amount of constitutional disturbance likely to be caused by the eruption of a mass, two, three, four or more times the size of such a tooth.

*Radicular Osteo-dentomata.*—Structurally these are of hard tissue, traversed by canals which contain blood vessels. The dentinal matter may be deposited in a globular form, from the vascular canals; tubules, resembling in their size and mode of ramification those of dentine, pass off and lose themselves indefinitely in the surrounding hard structure.

No odontomes belonging to this group have been described in the human subject, and as far as our present knowledge extends, the only mammal in which they have been described is the elephant.

It is necessary to draw attention to those large masses of osteo-dentine, which occur so frequently in the pulp of elephants' teeth, not only in the tusk, but also in the molars. These masses of secondary or osteo-dentine must not be regarded as odontomes, but as the direct result of inflammation of the pulp.

Small nodules of osteo-dentine are of frequent occurrence in the pulp of healthy human teeth, and excite but little surprise. It is only their bulk which causes wonder, when they occur in the pulp of elephants' teeth.

Similar formations of osteo-dentine occur normally in the teeth of the sperm whale and in the grampus.

D. *Aberrations of the whole Tooth-germ.*

*Composite Odontomata.*—This is a convenient term to apply to those hard tooth tumours, which bear little or no resemblance in shape to teeth, but occur in the jaws, and consist of a disordered conglomeration of enamel, dentine, and cementum. Such odontomes may be considered as arising from an abnormal growth of all the elements of a tooth-germ—enamel-organ, papilla, and follicle.

Up to the present time I have found no such odontomes in the lower animals, all the recorded cases having occurred in man. A typical odontome of this group is the one described by Mr. Heath, as occurring in the lower jaw of a young lady aged eighteen. The clinical history in this case is very instructive, and the reader is referred to the original account of it in the Clinical Society's "Transactions," vol. xv.

In the same category may be placed the odontome dislodged by Professor Annandale from the jaw of a young woman aged seventeen, who never had any molar teeth in the left lower jaw. It weighed 300 grains. It consisted of dentine and osteo-dentine, capped by enamel.

Not only is this class of odontomes composite in that the tumours comprised in it originate from all the elements of a tooth-germ, but they are composite in another sense. In the majority of cases the tumour is composed of two or more tooth-germs indiscriminately fused. But they differ from the cementomata containing two or more teeth, from the fact that the various parts of the teeth composing the mass are indistinguishably mixed, whereas the individual teeth implicated in a cementomata can be clearly defined.

E. *Anomalous Odontomata.*

In this section we must consider a few very remarkable cases. The first was placed on record by Mr. Tellander, of Stockholm; the details are as follows:—

A female, aged twenty-seven, applied to him in consequence of an attack of inflammation of the right upper jaw, due, as she supposed, to the presence of the roots of a temporary molar. The temporary teeth, so far as was known, presented nothing unusual, and were shed and replaced by the permanent set, except that on the right side of the upper jaw, the first molar, the two bicuspids, and canine failed to appear. The spot where these teeth should have been became at the age of twelve the seat of hard painless enlargement. When the patient applied to Mr. Tellander there was a free discharge of pus from this spot; some stumps were removed, and carious bone detected. Subsequent examination showed that enclosed within this carious bone was a cluster of minute teeth. There were nine single teeth, each one perfect in itself, having a conical root with a conical crown tipped with enamel; also six masses built up of adherent single teeth. The denticles presented the usual characters of supernumerary teeth. About a year afterwards a tooth was found making its appearance in the spot from which the host of teeth was removed.

A similar case has been recorded by Sir John Tomes.

A third example of this remarkable condition has been recorded by Professor Windle and Mr. Humphreys, in the "Journal of 'Anat. and Physiology,'" vol. xxi. The case occurred in the practice of Mr. Sims, at the Dental Hospital, Birmingham. The tumour was found in the mouth of a boy aged ten years. It was found that neither the deciduous nor permanent right lateral incisor or canine had erupted. The space thus unoccupied was filled by a tumour with dense unyielding walls which occasioned no discomfort. On opening this cyst forty small denticles of curious and irregular forms were removed from the interior.

Mr. Heath records briefly a case of a similar nature in a boy, aged four years.—*Odontological Society, Dental Record.*

